# Contingent Relations: Migrant Wellbeing and Economic Development in Rural Manitoba

**DOI:** 10.3389/fsoc.2021.596939

**Published:** 2021-02-15

**Authors:** Catherine Bryan

**Affiliations:** School of Social Work, Dalhousie University, Halifax, NS, Canada

**Keywords:** migrant labour, manitoba, rural economic development, migrant wellbeing, service and hospitality

## Abstract

Drawing on ethnographic fieldwork conducted in rural Manitoba and the Philippines, this paper uses the example of the small town of Douglas, which since 2009 has been home to a small Filipino community, as a tenuous counter-point to the accounts of exclusion that dominate the scholarship on Temporary Foreign Labour in Canada. This paper draws on ethnographic research conducted in Manitoba with the region’s newest immigrants—those recruited to ensure the viability of the new, diversified rural regional economy, and more specifically, the tourism and hospitality sector, established in the 1970s. In 2009, unable to meet its labour needs regionally, a local hotel began recruiting temporary foreign labour. By 2014, the Hotel had recruited 71 workers from the Philippines, most of whom arrived through Canada’s Temporary Foreign Worker Program; others having arrived through the province’s immigration scheme, the Manitoba Provincial Nominee Program (MPNP). A reflection of the ubiquity of globalized Filipino migration, the well-being of these workers had long been informed by economic development in the Philippines and the centrality of international labour mobility to that state project. What emerges from the data is a simultaneous acceptance and contestation of the conditions of transnational family life, and moreover—reflecting the focus of this special issue—the extent to which migrant well-being shifts in accordance to labour mobility regimes responsive to development. Migrant workers and their families are implicated in these connected, yet differently motivated, state projects. And while particular narratives concerning their contributions come to be valorized and even celebrated, their mental, physical, affective, and relational well-being is often over-looked by those who benefit from their labour and mobility. Of equal importance is the provincial state’s participation in this process through the provision of permanent residency to existing and in-coming migrants. While this benefits individual families, it does not inherently challenge the logics of neoliberalism; rather, drawing on its nuances, it create new possibilities for capital accumulation and exploitation, while offering some protection for select families who are willing and able to abide by the terms established by their employer and the Manitoba state.

## Introduction

Tracing the exploitative potential of temporary labour migration and its value for economic and social development in both sending- and receiving-sites, this article highlights a somewhat anomalous immigrant labour recruitment strategy undertaken in collaboration by rural employers and the Manitoban government. It does so as a means of elaborating the contingent relationship between migrant wellbeing, rural economic development, and the partial or potential social inclusion increasingly on offer by the Canadian state, via Provincial Nominee Programs. While this inclusion emerges as a possible win for employers and workers alike, in a global context characterized by high levels of migrant vulnerability, dependence, and precarity, it also shrouds the underlying exploitative logic of labour migration schemes historically and in the contemporary neoliberal moment. Furthermore, it obscures how the potential of permanency can be deployed by employers and government alike to anchor low-cost labour in place as means of ensuring the profitability of small-scale capitalist enterprise. More precisely, this article draws on the first 5 years of migrant labour recruitment by a small, rural hotel and conference centre in the town of Douglas, Manitoba. 3.75 km-squared with a permanent population of approximately 1700 people, the town is a thoroughfare for most: truckers, mobile construction crews, regional travelers, and tourists arrive with some frequency at the Hotel or its adjacent amenities, staying for the night, a few days, or—in the case of construction crews—a few weeks. Like many rural centres across the Canadian prairies (Moss et al., 2010; Nakache and Blanchard, 2014; Bucklaschuk, 2015; Wright et al., 2017), the labour required of meeting the needs of these temporarily mobile people has fallen to a largely migrant workforce, comprised predominantly of Filipino workers, though more recently, workers from the Caribbean (Bryan 2019a; Bryan, 2019b).

A reflection of the ubiquity of globalized Filipino migration, the well-being of these workers and their families has long been informed by economic development in the Philippines and the centrality of international labour mobility to that state project. Indeed, all of the migrants interviewed had protracted experience with migration and had long relied on overseas employment to meet their families’ social reproductive needs. What emerges from the data is a simultaneous acceptance and contestation of the conditions of transnational family life, and moreover—reflecting the focus of this special issue—the extent to which migrant well-being shifts in accordance to labour mobility regimes responsive to development. These regimes are, at once, those of the Philippines, which seeks to redress social and economic instability in that country through labour export; of Canada, which aims to secure precarious and dependent migrant (read temporary) labour for local labour markets; and of Manitoba, which seeks out new resident-workers to safe-guard rural economies against the volatility of neoliberal transition within traditional sectors—notably, agriculture, mining, and logging. Migrant workers and their families are implicated in these connected, yet differently motivated, state projects. And while particular narratives concerning their contributions come to be valorized and even celebrated (particularly in the Philippines and Manitoba), their mental, physical, affective, and relational well-being is often over-looked by those who benefit from their labour and mobility. Embedded in protracted and transnational histories of colonialism and capitalism, migrant well-being is an affective and material state that is always in process, responding to the changing conditions and requirements of capital accumulation across manifold sites. Indeed, migrants abide by the norms of “transnational Filipino family”, while actively seeking out alternatives. For the migrants at the centre of this analysis, the desire for a family-life “in place” has prompted a unique relationship with their employer who draws on the networks of its Filipino workforce in the recruitment and retention of labour.

Taking as its starting point state-affect discourse concerning health and wellness, the article begins with a discussion of migrant wellbeing. Following a description of the study’s methods, the first substantive section unpacks migrant wellbeing in relation to legal status. Whereas permanent residents and immigrant citizens are able to avail themselves of state-provided health and wellness services, temporary foreign workers are largely excluded. At the same time, they are often centred in emotionally driven debate concerning the wellbeing of “local” labour. Importantly, “local” labour—or rather its shortage—is often at the core of employer demands vis-à-vis the state and the Temporary Foreign Worker Program. Across sectors and regions, the argument made by employers is that due to a lack of available local resident-workers, they require a policy mechanism through which to recruit non-resident workers. As elsewhere, in Manitoba’s rural tourism and hospitality sector, this argument is frequently grounded in well-known and accepted tropes of rural depopulation. Less on offer are the structural reasons for depopulation, and more significantly, the unwillingness of local labour to take up employment in the sector. Indeed, in conversation with long-standing local residents, including those who make up the Hotel’s local clientele, strong preference for the working conditions, schedules, and wages of construction, transportation, and care labour is often expressed.

Elaborated in the second section, the region’s economy transformed in the early 1970s. Integrated more fully into international markets, increasingly mechanized, and requiring staggering amounts of capital investment, agriculture was no longer a viable livelihood option for the majority of rural residents. In turn, working with government, local business sought to diversify the economy. Intended to establish new revenue streams *and* local employment opportunities, tourism surfaced as a key strategy. However, despite the creation of new jobs, local resident workers have tended to seek out employment elsewhere—an outcome of the low-wages and low-status often associated with service and hospitality work. Thus, the sector, which was established in the 1970s and expanded in the 1990s, has had to rely on migrant workers to remain profitable. The focus of section three, the labour supporting the on-going economic development of Manitoba’s rural tourism sector has not been “local”, but rather “global” labour that is set in motion through the efforts of the Philippine states on the one hand, and the Canadian state on the other.

Recruited to ensure the viability of Manitoba’s new diversified rural regional economy, and more specifically, the tourism and hospitality sector, the first wave of these workers arrived between 2009 and 2014 and were deployed across the Hotel’s various departments. All but one of the initial 71 migrants recruited by the Hotel would become permanent residents—with a large majority becoming Canadian citizens within 5 years of the completion of the study (by 2019). Representing a key distinction between migrant labour recruitment in other Canadian jurisdictions and globally, the Hotel deploys permanency—made possible through Manitoba’s immigration scheme, the Manitoba Provincial Nominee Program—as a labour recruitment and retention strategy. From this labour migration scenario, section four provides several ethnographic examples of Filipino family life and migrant wellbeing as constituted and re-constituted through sub-national immigration policy in Canada that seeks to make permanent once temporary foreign workers. Central to this discussion is the contentious and contingent status of “wellbeing” as an analytical category and desired outcome, particularly as applied to migrants, whose wellness in Canada often hinges on legal status. Even more broadly, when situated in the protracted and transnational histories of colonialism and capitalism, migrant wellbeing loses any solid or consistent quality. Providing the central analytical contribution of the paper, the final discussion section posits migrant wellbeing as an affective and material state that, responsive to the changing conditions of capitalist political economy across manifold sites and the requirements of capital accumulation overtime, is always in process. Reflective of the pervasive inequality that undergirds global labour migration regimes, this remains true even where permanent residency is on offer and where employers and states appear invested, on some level, in migrant “wellbeing”. Importantly, then, for this work, while “wellbeing” is used as a proxy for the positive emotional, relational, and physical health outcomes of permanency as anticipated and subjectively experienced by the Hotel’s migrant workers, it remains tied to state development projects that undercut the possibility of “wellness” for most migrants globally. As argued, migrants are responsibilized by both sending- and receiving-states for their own wellbeing in contexts characterized by high levels of state-sanctioned exploitability. An exception that proves and reinforces the rule, those at the centre of this analysis were able circumvent some of the vulnerability ad precarity endemic in most labour migration scenarios, but yet, remained bound to the logics and demands of temporary forms of labour mobility.

## Methods

As Wolf illustrates across his scholarship, rather than “encompassing the whole world in a homogeneous field of effects”, capitalism generates “variability and differentiation not only through its combination with other modes [of production] but also in the very course of its own operations” (Wolf, 2010, p. 303). Following from this, inequality and stratification—that is, the ordering of inequality—are generated by, and generative of, the capitalist mode of production. Moreover, they are necessary mechanisms of accumulation as value is mobilized and transferred between sites. The arrival of Filipino workers in Douglas reflects such a transfer in the form of labour power. At the same time, when we account for the social geographies and political economies of each site and the ways in which both have been integrated in complex ways into the circuits of global capitalism over time, the story of Philippine workers in Douglas offers something far more complicated: insight not only into the arrival of 71 people in Douglas, but into the coalescing and contingent relationships and dynamics that over time, have produced the conditions conducive to that arrival. In the ethnographic tradition of Wolf (2010), and like other anthropologists engaged in studies of political economy (Carrier and Kalb, 2015; Kasmir and Carbonella, 2017) and migration (Mahler and Pessar, 2006; Brettell and Hollifield, 2014), this article elaborates the connection between migrant wellbeing and these multi-sited relationships and dynamics as they relate to economic development in rural Manitoba.

Data for this analysis was collected by the author between 2012 and 2014 with 71 migrant workers recruited to work in Manitoba’s new, diversified rural regional economy, and more specifically, the tourism and hospitality sector, established (though tenuously) in the 1970s, and their non-migrant kin in the Philippines. In total, 131 in-depth life history interviews were conducted in Manitoba and sites across the Philippines. These interviews focused on the labour mobility histories of 21 transnational family groups (totalling 81 individuals: 21 migrants and 60 non-migrant kin) and 50 additional migrants in Douglas whose families, in the Philippines, did not participate in the study. Fieldwork was conducted in four installments over a 2 year period. At each stage, different kinds of data were gathered. In the first instance (December 2012), in-depth interviews were conducted with the Hotel’s Philippine workforce. At the time, only 44 foreign workers had been recruited by the Hotel. Of these, 43 had arrived through the Temporary Foreign Worker Program since 2009, and one had arrived through the Manitoba Provincial Nominee Program. During this first visit, in-depth interviews were conducted with 25 of the Hotel’s Filipino workers, as well as with one TFW who had initially arrived (2011) in Manitoba to work at an industrial hog barn, 1 h north of Douglas, but was currently employed at the local veterinary hospital. These interviews were focused on each participant’s labour migration history, reasons for seeking out overseas employment, work experience at the Hotel, and transnational kinship practices.

Conducted in the Philippines, the second phase of research was designed to capture the lives and experiences of those “left behind” and “in waiting”—non-migrants who came to be integrated and implicated in transnational strategies of livelihood and reproduction through the labour migration of a spouse, a parent, a child, or a sibling. In the Philippines, 60 non-migrant family members were interviewed. The third and fourth stages of research were conducted in Douglas. At each stage of research conducted in Douglas, interviews and follow-up interviews were conducted with relevant stakeholders: Hotel management, the regional settlement workers, and a local economic development officer. During each phase, interviews were supplemented with participant observation in both Douglas (at the Hotel) and in the Philippines. Additional in-depth interviews were done with Hotel management, regional economic development officers, the regional settlement services provider, and several long-standing local business owners. Archival research was conducted in both the regional economic development office and the provincial archive (located in Winnipeg). Here, the objective was to better understand state practices and adjoining discourses vis-à-vis development overtime.

## Migrant Wellbeing

The popularization of “wellbeing” as a research focus and more recently, as a policy objective, reveals a highly contested concept (Sointu, 2005; Schwanen and Atkinson, 2015). Critics of the term have also pointed to its vulnerability to commodification and neoliberal appropriation (Esposito and Perez, 2014). Under these conditions, wellbeing becomes an exercise in self-management, whereby individuals avail themselves of privately provisioned goods and services in the pursuit of a “wellness” bound to middle-class conceptualizations of the “good life” (Firth, 2016). Reading together the growing prevalence of resiliency narratives (Howard et al., 1999; Fletcher and Sarkar, 2013; MacKinnon and Derickson, 2013), the retrenchment of the welfare state (Starke, 2006), the emergence of workfare policies and programs (Peck, 2001; Gazso and McDaniel, 2010), scholars argue that the state’s promotion of wellness is more broadly a strategy intended to ensure employability and the capacity of labour to withstand precarity and uncertainty. This strategy, however, is not uniformly applied to all workers. Migrants—particularly those whose legal status is tenuous or who are undocumented—are largely excluded from such discourses. In Canada, this exclusion is formalized through the restrictive parameters of the Temporary Foreign Worker Program that, for most migrant workers, prohibit family reunification and limit access to health care.

If temporary migrant workers are excluded from neoliberal state affect discourse in the site of employment, they are often central to emotionally driven narratives concerning “local” labour and its relative value. For example, between 2012 and 2014, following several highly publicized tragedies and controversies (CBC, 2011; CBC, 2012a; CBC, 2012b), the Temporary Worker Program became the subject of considerable public and political debate. While some of this focused on the exploitative conditions engendered by the program (CBC, 2013; CBC, 2014a; CBC, 2014b; CBC, 2014c), the perception that citizen- and resident-workers were being excluded from labour markets as employers became reliant on temporary foreign workers dominated public discussions (CBC, 2014d; CBC, 2014e; CBC, 2014f; CBC, 2014g). Despite some attention paid to working and living conditions in Canada, much of the media coverage during this period spun those realities to highlight the value-added offered by migrant labour. Reduced to units of labour, the aspirations and health of migrants are frequently regarded by employers and governments as tangential to the operations of capital accumulation. In contrast, drawing on the experiences of the migrant workers at the centre of this analysis, migration is, for many, a high-stakes investment in the material and social well-being of family and kin. In a context that mostly overlooks the migrant well-being, not only does the project of migration require considerable planning, sacrifice, and risk-taking, it often demands intensive emotional regulation (Bryan, 2017; Bryan, 2018; Hochschild, 2000; Hochschild, 2012). Given shape by what Tungohan (2018) calls ideational factors—the ideological narratives and cultural scripts that explain and rationalize wide-spread labour migration from the Philippines—this affective labour is used to navigate and survive the often times toxic character of life and labour for migrant workers in Canada.

Over the last ten years, a considerable scholarship has emerged that explores and documents the subjectively experienced physical, emotional, mental, and material wellbeing of migrants or their kin (both migrant and non-migrant), either in the context of resettlement or the country of origin (Gushulak et al., 2011; Sptizer, 2011; Wright, 2011; Berry and Hou, 2016; Campos-Flores and Dabrowska-Miciula, 2018; Caxaj and Diaz, 2018). Largely bifurcated, this academic literature corresponds either to landed immigrants (those who arrive as permanent residents) or migrant labour (those who arrive with a temporary designation), with government intervention focused largely on the former. In regard to permanently settled newcomers, the research effectively highlights a range of structural barriers related to language proficiency (Pot et al., 2020), racism and xenophobia, credential recognition, and employment (Aycan and Berry, 1996). These are seen as negatively impacting wellbeing in so far as they limit access to meaningful employment and supportive services (such as healthcare), and they are seen as having inter-generation effects (Berry and Hou, 2016; Hadfield et al., 2017). Thus, in this literature, the function of immigration policy as generating vulnerability and precarity is not centred; instead, the social, cultural, and economic context that condition the experience of integration is prioritized, as well as the intersecting forms of discrimination and oppression informing that context. When taken up by the state, these insights filter into a gamut of services and programs intended to equip newcomers with the skills required to navigate less-than-welcoming environments, thereby—in principle—maximizing their wellbeing.

In addition to the scholarship on immigrant wellbeing, an important body of literature captures the mental and physical health outcomes associated with temporary labour migration (Bernhard et al., 2007; Fuller and Vosko, 2008; Bahn, 2015; Caxaj and Diaz, 2018; Salami et al., 2015). While many of the experiences are similar, mapping onto social inequity, racism and other forms discrimination in Canada, for those who arrive as temporary foreign workers, wellbeing may be further adversely effected by exposure to workplace injury and illness, and compounded by an inability to access health care (following the restrictions of the Temporary Foreign Worker Program) (Hennebry et al., 2016; Preibisch and Hennebry, 2011). For some workers, notably those in agricultural, this is further exacerbated by inadequate housing and sanitation, a lack of clean water, and proximity to chemicals and pesticides (Magalhaes et al., 2010; Helps, 2020). In addition to physical outcomes, poor mental health outcomes are also common. Here, poor and exploitative working conditions coalesce with challenging work relationships, communication barriers, loneliness, and separation from significant relations and supports. Amongst these features of migrant life, the fear of deportation and repatriation—an outcome of state policy, is often the most destabilizing, as it taps into, not only insecurities and inequalities that exist in the site of employment, but those endemic in the sending-state and affecting of non-migrant kin (Walia, 2010; Perry, 2012; Nakache, 2018; Perry 2018). Different from the literature on permanently resettled migrants, this scholarship focuses considerably on the effects of immigration policy in Canada (Oxman-Martinez et al., 2005) and emigration policy in the Philippines. While not always considered in tandem, both are regarded as directly and negatively impacting the physical and mental health, and wellbeing, of migrant workers. Running adjacent, is a significant critique of the lack of state-funded services for this cohort of workers once in Canada (Dabrowska-Miciula and de Lima, 2020). Here, in the absence supports (such as healthcare), migrant workers vend for themselves or rely on the (inconsistent) benevolence of their employers.

Despite important consideration of the policy that facilitates labour migration and determines its conditions, less visible in this scholarship are the adjacent policy regimes that generate the purported need for temporary migrant labour in the first place. In other words, the focus tends to be on the individual or shared outcomes of migrants as they follow from the restrictions of—in the case of Canada—the Temporary Foreign Worker Program. And yet, the TFWP is, itself, a response to a set of conditions and processes related to economic development that are historic, contemporary, highlight local, and transnational in character. Importantly, then, migrant wellbeing is relational; it responds to opportunities and obstacles that are situated at the intersection of local, national, and international immigration regimes that collate with similarly scaled labour markets, economies, and state development projects. Equally absent from this literature is consideration of effects of the transition or potential transition to permanent residency. While permanent residency status is held out as a solution to the damaging effects of temporary labour programs (Nakache and Blanchard, 2014), with some notable exceptions (Polanco, 2014; Bonifacio, 2015; Tungohan et al., 2015; Polanco, 2016; Bryan, 2019a), little has been published on the actual implications of transitioning to permanency for temporary foreign workers and their kin. In addition to redressing this, this paper also illustrates the ways in which the “taken-for-granted” good of permanency can also be harnessed by state and capital to hold migrants in place, and to duplicate hierarchies and structures of accumulation that benefit employers. Thus, while a laudable objective, wellbeing, when bound to processes that allow some workers (and not others) to transition to permanent residency, merely reinforces the individualism of neoliberal wellness

## Local Hospitality; Global Labour

In rural Manitoba, where the population is sparse and out-migration common (Silvius and Annis, 2007), the challenges of labour recruitment and retention are frequently rehearsed by employers and local government. This challenge—or the perception of it—accelerates in the service and hospitality sector that, historically feminized, is characterized by precarity, low-wages, and low-status (Bryan, 2018). In the region, service and hospitality is closely aligned with the region’s tourism sector, which emerged as an economic diversification strategy in the late 1960s and accelerated in the 1990s. Intended to redress and sustain emerging trends in agriculture, specifically, consolidation and intensification, tourism, it was held by local and provincial officials, would ensure the on-going viability of agricultural production, albeit in a modified form, through the creation of new economic opportunities, and most importantly, new local jobs. Tourism, in other words, was to run adjacent to agriculture, bolstering the rural economy and creating the conditions necessary for agricultural production to survive its neoliberal reorientation.

Beginning in the 1960s, the economic structure of industrialized countries, like Canada, began to change at an increased rate, prompted by the growing internationalization of capital markets, higher levels of transnational investment, and the gradual emergence of nascent neoliberal doctrine and practice (Ghorayshi, 1990). Reflective of a larger project of rural economic development, which took as its starting point the restructuring of the agricultural sector, tourism was to redress *and* sustain emerging trends in agriculture—notably, consolidation and intensification. This two-pronged objective would be achieved through the diversification of the rural economy, which, in turn, could continue to support agricultural production. Much like elsewhere in the province, then, tourism in the region is not seen as cure-all to rural economic decline; rather, as Ramsey and Everitt (2007) explain, it is regarded as a “smokeless industry”, offering additional and sustainable economic security for rural communities. An economic additive, tourism has been integrated into existing sectors of the rural economy—wilderness, forestry, hunting and angling, sports, arts, culture, and heritage. Officials, and those involved in the sector, stress the potential manifold positive outcomes for local businesses and local tax bases. These benefits are generated by visitors, and by those who work in the industry, and it is anticipated, will create and sustain local employment, bolstering the rural economy, and safe-guarding the future of rural-life. Following this logic, recreation and tourism have come to significantly inform the social and economic agendas of the region, even as agricultural production and its off-shoots remain dominant (Kulshreshtha, 2011).

Reflective of the tertiary sector more generally (Jamal and Getz, 1999), however, the positions offered by the region’s tourism sector, while localized, tend to be low-salary and low-status. They are often temporary and seasonal in nature, and even those that are permanent and year-round remain tethered to the seasonal ebbs and flows of the region’s primary tourist attraction, the Ski Hill. Workers at the Hotel, for example, retain their positions during the off-season, but their hours may be negatively affected depending upon occupancy. These conditions serve as stumbling blocks to the recruitment and retention of labour, and following from them, the Hotel has had to look elsewhere for labour. Importantly, then, for the Hotel (which requires a staff of approximately 110 workers year-round), the gradual diversification of the rural regional economy has run parallel to a number of developments: the institutionalization of labour export in the Philippines, the establishment and eventual expansion of the Canadian Temporary Foreign Worker Program (TFWP), and the more recent parallel, though not always harmonious, provincial interest in immigration. Filipino migrant workers in Manitoba find themselves at the intersection of these three labour mobility regimes. Each embedded in larger state projects of development, they dictate the terms and conditions of migration, and as such, are intrinsically connected to and affecting of migrant well-being.

That overseas labour migration might benefit local, migrant-sending, economies is a position long held by states and non-governmental organizations. In the case of the Philippines, this outcome has been pursued through a complex institutional lattice work consisting of various government departments and agencies, and a state-regulated private sector (Bello et al., 2005). As a result, the Philippines is one of the world’s top exporters of labour in the world (Ball, 2006; Tyner and Donaldson, 1999; Solomon, 2009; Agbola and Acupan, 2010). Predicated on a long history of internal and regional labour mobility (De Jong et al., 1983; Barber and Bryan, 2012), the country—under the Marcos regime—formerly instituted labour export in the early 1970s (Tyner, 1999). Reflected in the 1974 Labour Code, all labour policies, including those related to labour export, were brought in-line with the country’s overall development goals. By the early 1980s, the country owed $21 billion dollars in foreign debt, and a set of structural adjustment programs were initiated by the state. Well-rehearsed in the academic and grey literature, rather than realizing its top-down redistributive objectives, structural adjustment would deepen inequality in the country. And as wealth and power became further concentrated in the hands of small elite, social and economic inclusion for a significant portion of the population remained illusory (Baggio, 2008; Rodriguez, 2017). In turn, the Marcos regime intensified labour export as a means of shoring up the Philippine economy. During this early period of labour export, the regime deployed two discursive formulations to justify the state’s involvement and intervention in overseas labour migration: the first stressed the inevitability of population movement in light of global disparities; the second, a discourse of sacrifice, suggested that individual Filipinos should submit to this natural phenomenon, sacrificing themselves for the good of the nation and in the service of their families (Tyner, 2009).

The current iteration of migrant subjectivity-producing and -reproducing discourse draws heavily on neoliberal doctrine concerning the inevitability of globalization, the neutrality of capital, and the primacy of the individual (Rodriguez, 2013; Rodriguez and Schwenken, 2013; Ortega, 2016; Polanco, 2017). Following from the ideological parametres of neoliberalism, the state would no longer assume a proactive position regarding migration; rather, it would simply manage what was already happening (Tyner, 2009). From this, a new state interpellation of migration and migrant identity emerged. According to it, “naturally adventurous” Filipinos were simply participating in the “cultural of migration” that had evolved in the Philippines (Guevarra, 2010). Empowered and independent, the new modern migrant “chooses” migration as a means of self-improvement and upward mobility (Guevarra, 2010; Guevarra, 2014). Migrants are, thus, fully rational agents, responsible for their migration and moreover, the conditions of their labour once abroad. Practically, this absolves government of any responsibility for migrant workers, while enabling the state to download the many of the costs associated with economic development onto its migrant citizens. Though often focused on earning capacity of migrants and their contributions to Gross Domestic Product via remittances (Bove and Elia, 2017), migration as development encompasses a wide range of anticipated practices and outcomes. These include human capital investment, investment in infrastructure (housing and community-based), as well as the transfer of technological and cultural norms (Clemens et al., 2014; Özden and Rapoport, 2018).

If Filipino workers are responsibilized for the conditions of their migration by the Philippine state, they are, following the parameters of the Temporary Foreign Worker Program, equally responsibilized by the Canadian state for their well-being once in Canada. Over the last thirty years, a significant body of critical scholarship has emerged in Canada, focused on the exploitative nature of the Temporary Foreign Worker Program (Fudge and MacPhail, 2009; Foster, 2012; McLaughlin and Hennerbry, 2013; Goldring and Joly, 2014; Beatson and Hanley, 2017; Horgan and Liinamaa, 2017; Strauss and McGrath, 2017). Drawing on Marx and grounding their analysis in the experiences, claims, and struggles of migrant and immigrant workers, Choudry and Smith offer a useful conceptualization of the condition of temporary foreign labour in the country. Destabilizing one of capitalism’s most enduring fallacies, they argue that the myth of “free” labour obscures the extent to which the Canadian economy has long relied on “exploitation through race, immigration status, and shifting forms of ‘unfree labour’” (Choudry and Smith, 2016). Revealed in an expansive body of academic and grey literature, the threat of deportation renders migrants extremely vulnerable to range of profit generating and/or accelerating practices (Basok, 1999). Two years after the expansion of the Low Skilled Pilot Project, the Hotel’s first Filipino workers were recruited, and by 2014, 71 of the Hotel’s 110 employees were migrants, and the majority had arrived through the Temporary Foreign Worker Program.

## Findings: Migrant Well-Being in Transition

As the Filipino state to draw on migrant resources in the service of economic development in the Philippines is contingent upon the production and reproduction of social identities conducive to the labour export, in Manitoba, the province’s Nominee Program facilitates the long-term retention of workers through the provision of permanent status in Canada to some temporary foreign workers. For temporary foreign workers in the food sector, however, the pathway to permanency is contingent upon geography, so that depending on where a worker is employed, the Provincial Nominee Program in question may (or may not) allow for permanent residency. Provincial Nominee Programs (PNPs) have been a feature of Canadian immigration since the late 1990s. Managed through a series of bi-lateral agreements between the provinces and federal government, the PNPs represent a redistribution of jurisdictional responsibility for the development and implementation of immigration policy. These bi-lateral agreements empower provinces to “nominate” individuals who meet their respective PNP criteria. “Nomination” refers to the final step in the PNP application process, whereby, having approved an application, the province “nominates” the applicant for designation to their jurisdiction. That person then completes an application for permanent residency which includes their nomination, submitting it to Immigration, Refugees and Citizenship Canada (IRCC)^1^. Final responsibility for selection—effectively approving of the nomination and granting permanent residency status—rests with IRCC, which is the sole authority for the issuance of admission visas in Canada (Carter et al., 2008). As permanency for the Hotel’s temporary foreign workers became a possibility and then a reality, their long-term livelihood strategies and projects of social reproduction shifted. In turn, their framing of and goals vis-à-vis family life and the hoped-for wellbeing of kin were re-directed, often in unexpected ways.

Drawing on several examples from in-depth interviews and ethnographic fieldwork in Manitoba and the Philippines, this section traces the shifting parameters of migrant wellbeing as informed by the migration-driven economic development strategies of both the Philippine and Manitoba states. It concludes with a discussion of the material, relational, and affective implications of the permanent residency on offer to TFWs via the MPNP, arguing that while permanency certainly redresses some of the vulnerabilities generated by global labour mobility regimes (sending- and receiving-) for those able to avail of it, thereby enhancing migrants’ experiences of wellbeing, it simultaneously reinforces those vulnerabilities for those who cannot, while obscuring the precarity and exploitation characteristic of international labour recruitment. For many of the Hotel’s earliest migrant workers (those recruited in 2009, 2010, and 2011), the potential of permanent residency status was relatively unknown prior to arrival. For example, Ester, who was one of the first two workers to arrive in 2009, expected to work the duration of her 2 year contract; if she was fortunate, she explained in 2012, it would be renewed for another 2 years. In other words, when she arrived, she planned to stay for 2 years, but hoped she would be able to remain in the province for four, after which—and in line with the TFWP’s four-in four-out rule^2^—she would look for work elsewhere, likely in Dubai where she had been prior to Manitoba. Regardless of where she ended up, Ester only anticipated contract work and she only expected to remain for a few years. Her family would remain in the Philippines, and she would continue to care for them, but always from a significant distance. Her life, as she imagined it, would be characterized by a permanent impermanence and by a separation from family and other significant relations she had grown to accept, if not completely tolerate. That said, as she explained, even as she knew she would have to leave Canada, she was very hopeful that she might find a way to stay. This sentiment was common amongst the first cohort of migrants at the Hotel. As Peter, who arrived early in 2010 explained, “even as we know we have to leave, many Filipinos would like to stay if they can.” Fortunately, several weeks into her position at the Hotel, management informed Ester that after a six-month period, she would be eligible to apply, with their support, for permanent residency status through the Manitoba Nominee Program. This possibility, previously unknown, dramatically altered Ester’s plans, as well as the short- and long-term direction of her life.

When I first met Ester, she was on maternity leave. Her application with the MPNP successful, she was waiting to hear back with confirmation regarding her permanent residency status. She expressed hope about the future and was eagerly waiting for her husband to join her. As we spoke, she opened her mail; her son’s Social Insurance Number amongst it, she congratulated the 9-month old as he tentatively made his way around the living room of the small house Ester rented from the Hotel. *What does this mean for you?* I asked, *for your family in the Philippines and your life moving forward?*

“For so many years, I planned to work abroad. I studied Hotel Management at college so I could find a job. First, I was in Dubair, and then I came to Canada. Of course, I wanted My husband is a nurse. He works in rural areas in our province. We want very much to have a big family, but it is hard to imagine doing that if I am here and he is there. Of course, I hoped we would someday live together, but when, we didn’t know… maybe (laughing) when I retire…

We are still waiting for my PR card and then from their [my husband] will be able to be with us…We will continue to support my parents and my husband’s parents from here, but we will be together, us and [the baby], and that will make things easier; less lonely.” (Interview, Manitoba, December 2012) My husband sees the baby on Skype and he was online for the birth. He has missed a lot. He wants to be here.

As with Ester, for the majority migrant workers at the Hotel, arrival in Manitoba followed a protracted trajectory, both personal and familial, of short-term, circular labour migration. Rooted in the Philippine’s history of labour export, most had already worked abroad and nearly everyone had immediate family who had done the same. Violet who arrived early in 2012 explains:

“My mother worked in Hong Kong for a family with two little girls for almost twenty years. We saw her every 3 years, maybe two. My dad doesn’t work very well; maybe, a small job here and there, but not enough for me and my siblings…I decided to pursue HRM (hotel and restaurant management) in college. I knew that if I did that I could probably work abroad. At first, though, when my mom retired, I found work as a welder in Korea, and then I was hired by the Hotel and came to Canada. Living away from them is hard; I especially miss my youngest brother, but it is what we Filipinos do. I am paying for his school now…” (Interview, Manitoba, December 2012)

Violet’s Mother Offers Additional Detail. “There was no work for me when my children were small. I found a job with a family in Hong Kong. They (my employers) were good people. I went back to the Philippines every 2 years, but it they said they needed me, maybe three. They had two little girls. I worked from them for 16 years, from the time Violet was little until the time she finished college. She is smart. I wanted her to go to school, [but] I didn’t want her to work abroad. But I was tired from working and I wanted to be in the Philippines as I got older. She was the only one of her siblings able to go, so she went. She takes care of her brothers now, and she sends money for the house. It is what we do for our families even if it means we are not with them.” (Interview, Cebu, March 2013)

Long exposed to the realities of overseas employment, for many at the Hotel, like Violet, separation from family and other important relations had become a near ubiquitous feature of life. “It is hard. People here (in Manitoba) always says they feel sad for us Filipinos, and it’s not that it’s not hard, but I am used to it. My mom worked away for most of my life; I could not think of a life where my family would be in one place” (Violet, interview, Manitoba, December 2012). Her mother’s presence only punctuating her childhood, adolescence, and early adult life, Violet had long lived with the material and emotional realities of temporary legal status. It was not what she wanted for herself, but in light of her family’s economic need, she felt it necessary. To that end she applied for positions in Canada and New Zealand: “I took the job in Manitoba because they said that I might be able to become a permanent Canadian resident; I knew I would live away from my family, but at least there would be some stability for me, and then because of that, for them.”

Emma applied for a position at the Hotel at the suggestion of a friend and former co-worker who had successfully secured work there. Older than both Ester and Violet, her decision was somewhat differently configured. Sitting with her husband at a small restaurant in Douglas she described to me how she had been very motivated to remain in the Philippines while her children were young. She worked long hours as a front-desk manager at a hotel, but she was able to spend her evenings and Sundays with them. When her youngest was 13 and her eldest nearly 16, she applied to the Hotel in Douglas. Successful in her application, she landed as a temporary foreign worker in 2010. Emma’s transition to permanent residency made possible a range of opportunities for herself, her husband, and her children. Once her nomination from the province was secured, her husband arrived on an open-work permit. Approximately 18 months later, with their permanent residency status confirmed, they returned to the Philippines to pick up their children.

While initially the plan had been to spend the remainder of her working-life abroad and likely separated from her family to meet their growing financial needs (corresponding largely to funding post-secondary education), Emma’s new status as a permanent resident altered that. “I was nervous, very nervous” she explained, “to be away from them. They [weren’t] children anymore, but we are so close; the decision was hard. The reality of not being close to them, even [harder]…I cried a lot during the first year because I thought: ‘this is life now’. I thought about the future and it wasn’t with them.” (Interview, Manitoba, December 2012) Things improved somewhat when her husband arrived; they were able to look forward together and they knew it would only be a matter of time before their children could join them. In the Philippines, Emma’s son and daughter redirected their short- and long-term plans toward Manitoba. Her son—the eldest of the two—took a bartending course to improve the likelihood of finding work with the Hotel. Her daughter, in turn, planned to enroll in grade 12 and while completing her high school education, to work as a banquet server, again at the Hotel. We first met in the Philippines as they were about to leave for Canada. Despite some worry about leaving friends and other close family behind, they spoke with excitement about their new lives in Manitoba and more importantly, about living with their parents again after 3 years apart.

In the barangay where Violet’s parents and brothers live, most of the neighbours are relatives, and each family unit has multiple members who work and send money from abroad. Similarly, within the social and familial networks of Emma and Ester, there are long-standing and persistent patterns of migration. These patterns correspond to those described in the academic and grey literature on Filipino labour migration over the last five decades. And yet despite, a considerable amount of permanent relocation amongst Filipinos more broadly^3^, with very rare exception relative to others in the social and familial groups, Violet, Emma, and Ester are alone to transition to a legal status in the site of employment that grants them access to more than the most basic social rights and entitlements. In this way, even as other challenges persist, their lives, overall wellbeing, and mental and physical health, are less determined by the exploitative potential and conditions attending short-term and circular forms of labour migration.

At the same time, permanency altered or solidified family dynamics and would determine or circumscribe the future mobility of members. Once permanent, Violet, for example, asked her brother to remain in the Philippines. Despite his interest in overseas employment, Violet’s long-standing support of the family positioned her as the primary decision maker where the care of their parents was concerned. She would continue to provide for them materially, but her brother would remain in the Philippines to oversee their care. Amongst the migrant workers at the Hotel it was relatively common that their mobility would engender a relative’s immobility. Equally common, however, the permanency of the Hotel’s once temporary migrant workers fostered new and previously unanticipated form of mobility for family. Beyond children and spouses, the Hotel’s migrant workers facilitated the arrival of cousins, aunts, uncles, and siblings, often leveraging their loyalty to their employer to secure their relative’s employment at the Hotel. Hiring and eventually supporting the MPNP applications of their worker’s relatives became a key labour recruitment and retention strategy for the Hotel. Once permanent, migrant workers are no longer obliged to remain with the employer who hired them through the TFWP. Offering employment and eventual residency to the family members of previously temporary migrants deters workers from resigning and represents not only a means of recruiting but also, retaining workers. For rural Manitoba’s fledgling service and hospitality sector, then, issues related to both labour and population (both of which are required by the rural economy more broadly) are resolved.

## Discussion: Migrant Wellbeing as Site of Accumulation in Rural Manitoba

As revealed in the vignettes offered in the previous section, while the migrants at the centre of this discussion abide by the norms of “transnational Filipino family”, they simultaneously and actively seek out alternatives. For the migrants at the centre of this analysis, the desire for a family-life “in place” prompted a unique relationship with their employer who draws on the networks of its Filipino workforce in the recruitment and retention of labour. The Hotel represents a particularly unique case in so far as their intentions and actions vis-à-vis its migrant workforce are self-interested and, concurrently, motivated by care for their employees. Consistently in conversation and in more formal interviews with the author, Hotel management reflected on the life-altering nature of permanency for their otherwise, chronically temporary, migrant workers. Here, management displayed an acute understanding of the hardships of labour migration. The transition to permanency was, in other words, seen as a win-win. Where the workers remained with the hotel, which was the expectation, the Hotel was able to attract, recruit, and retain a more permanent workforce. In turn, those who had arrived to work on a temporary basis—their short- and long-term livelihood strategies once bound to the turbulent dictates of global labour import and export regimes, were able to settle permanently and reunite with friends and family. This, it was regarded correctly by Hotel management as significantly and positively impacting the wellbeing, both mental and physical, of their migrant workforce.

In these ways, migrant wellbeing is tethered to the systems that produce labour mobility and to the social, economic, and ideological structures in both the sending- and receiving states that determine the conditions under which that mobility is realized and experienced. Migrants’ are simultaneously subject, then, to the potentially exploitative practices of employers that generate mental and physical ill-health; the parameters of receiving-state policy managing migration that separates them from kin and limits their ability to access appropriate and often necessary health care; and to the social and economic inequalities that initiated their mobility in the first place. Restricting viable local employment opportunities, these inequalities are mobilized by sending-states in their efforts to bolster economic development through labour export. While simultaneously, they are capitalized on by receiving-states and employers as they seek to secure profitable forms of labour. Importantly, for the migrants at the Hotel in Manitoba, some of this is tempered by the transition to permanency. Being permanent means less dependency on their employer and therefore a greater capacity to contest problematic work dynamics and conditions. It also means a greater ability to access health care services albeit it, in the context of rural Manitoba, these may not meet all of their needs—particularly around mental health. Permanency also offers the opportunity to be reunited with family, and in this way, it redresses the loneliness, worry, and emotional toll that can accompany labour migration. Sheila who arrived in 2012 describes the resolution of this set of feelings:

You remember [Catherine] when we first met (December 2012) and I had just arrived? We had coffee in the restaurant. I was so worried for my children. Proud of myself for taking care of them and their school, but so scared all the time that something might happen to them, especially the youngest, while I’m here…Since they arrived in Manitoba (spring 2014), there are new challenges, but at least they are here. I don’t have to worry about them in the same way I did before. [Laughing] I sleep much better. (Interview in Douglas, June 2014)

Sheila had arrived 3 months before our initial meeting. Although she belonged to an extensive transnational family network, with relatives across the United States, she had never lived or worked abroad. Prompted by a desire to pay eldest child’s university fees and to be more independent, Sheila was—when we first met—just settling into the routine feelings of separation. She was motivated to work and to send money home, but the evenings were difficult and the nights—often spent talking online line or texting with her children and husband—were long. Migrant workers in Douglas, as elsewhere, mitigated the emotional and practice difficulties associated with separation from family through provisioning work and near-constant contact through communication technology. While mostly effective, these practices were only ever partial, and coupled with the 12 h time differences, most continued to described feeling very far from loved ones. Over the course of this study, however, those feelings and the practical challenges of connection dissipated for most as family arrived.

Despite some criticism of the PNPs broadly (Dobrowolsky, 2011; Dobrowolsky, 2013; Dobrowolsky et al., 2015; Bryan, 2019a), the Manitoba program is typically held in high regard. In large measure, researchers of migration, policy makers (in the province as elsewhere in Canada), and immigrants themselves regard the MPNP as a success. Within the first ten years, it more than doubled Manitoba’s share of immigration, from 2% to 4.6% of landings. Moreover, the program has effectively attracted newcomers to rural areas, accounting for regional growth in housing sales and boosts in local business. Prioritizing these outcomes, the narrative offered by the province stresses the positive economic impact of permanent population growth (CBC, 2018; Goertzen, 2019; Government of Manitoba, 2020). Here, “nominees” (the informal label attached to immigrants who arrive through the Program) are the new workers, new consumers, and new tax base of the regional rural economy. They bolster local labour markets, contribute to local industry, and participate in local social institutions. In Douglas, such contributions to rural regional development are seen across a range of sites and sectors, as well as in the development of a small retail sector that offers Filipino products and money transfer services. At the Hotel, the question of labour is largely resolved, while a new Filipino customer-base (comprised of staff and their families) has emerged. At the same time, adjacent employment sectors in service and hospitality, as well as in agriculture and construction have been able to hire these new residents, their children, and their recently arrived spouses.

Often overlooked is that the dual labour/citizen subject required of rural economic development is often claimed from the inward flow of *temporary* workers to province. Corresponding to the study period, TFWs working in Manitoba frequently transitioned to permanent residency through the provisions of the MPNP, which allowed those with a full-time, permanent job offer to become permanent residents. As a result, many rural employers—including the Hotel—came to rely on the Program to recruit and retain their migrant workers. Largely absent from political discourse and the campaigns of regional economic development offices vis-à-vis immigration is acknowledgement of the initial conditions, restrictions, and forms of exploitation experienced by many rural nominees. The depth of this omission becomes all the more significant when read against the history of labour export in the Philippines and the reasons why Filipino workers seek out temporary overseas employment in the first place. Citing saturated labour markets, low wages, ineffectual government, social insecurity, and the near-constant threat of environmental disaster as motivating their migration, those at the Hotel offer good reason to seek out overseas employment. Through their labour migration, education is supported; the necessities of life are provided for; livelihood projects are sustained; concrete houses are built—survival is ensured, and life is safe-guarded. Thus, the profound connection between employment in Canada and survival in the Philippines deepens the dependency created by the Temporary Foreign Worker program, heightening the consequences of impermanence and importantly, making the possibility permanent residency all the more salient. Obscured, then, are not only the exploitative conditions present in Manitoba, but the extent to which the rural development in the province relies on and reproduces the profound social and economic inequalities upon which the global political economy of circular, guest, and temporary forms of labour migration are founded.

## Conclusion

Forty years after the neoliberalization of Canadian agriculture, tourism is a central, if somewhat tenuous, feature of rural Manitoba’s economy. The Douglas Hotel is the sector’s largest employer, but importantly, most of its workforce are not “local” in the conventional sense; rather, nearly three-quarters are migrants. In a very real way, then, and as elaborated above, the local success of the region’s tourism sector is predicated on processes initiated and enacted not only within Manitoba, but within a broader global political economy connecting the province and the Hotel to the Philippines. The wellness of this cohort of migrants, then, was produced, reproduced, and re-directed at the intersection of multiple state development projects, and largely informed by the ways in which employers mediate these projects through their recruitment and retention practices. And yet, their arrival and eventual settlement in Manitoba offers a tenuous alternative to much of the scholarship on temporary labour migration globally, and more specifically in Canada. In this literature, the conditions promoted by guest worker programs are routinely and necessarily rehearsed (Fudge and MacPhail, 2009; Nakache and Kinoshita, 2010; Sharma, 2012; Binford, 2013; Strauss and McGrath, 2017; Weiler et al., 2017). Often prioritized in this critique is the extent to which the value of temporary migrant workers is tied to their exclusion from social, economic, and political life within the host country. The Canadian example, however, offers a series of potential counterpoints to this narrative, following largely from the potential of permanent residency offered by a growing number of sub-nationally developed and implemented immigration schemes, referred to as Provincial Nominee Programs (Morrish, 2004; Carter et al., 2008). Indeed, where permanency is possible and supported, the exploitative tendencies of the Temporary Foreign Worker program are effectively undercut. Workers are no longer dependent on their employers, nor are they vulnerable to the threat of deportation. The transition to permanent residency also brings opportunity for family reunification, and for a life (often highly sought after) lived more in place (Bryan 2019a).

For those at the centre of this analysis, wellbeing—as a subjectively experienced and understood state—had been long informed by the centrality of international labour mobility to economic development in the Philippines. Indeed, like for so many globally as illustrated in a rich and in-depth literature, all of the migrants interviewed had long-standing experience with migration and had long relied on overseas employment to meet their families’ social reproductive needs. Once in Canada, these familial projects of livelihood and reproduction became dependent on the opportunities available there. Initially, this meant employment: temporary, short-term, and infused with the precarity and vulnerabilities of guest labour programs more generally. Eventually, however, it meant permanent residency—an outcome made possible by Manitoba’s provincial immigration scheme. In Manitoba—as revealed in local news media coverage and in conversation with Hotel management—local employers are quick to highlight the benefits of permanency, focusing primarily on family reunification, migrant access to health care and other state-provided services, and the localization of migrant spending. All of which, in turn, yield tangible economic benefits. Here, the logic goes that through family reunification, remittance sending decreases as migrants turn their attention to establishing themselves and their children in Manitoba. Moreover, workers experience less stress, have better health outcomes, and as such, are able to be more productive. Absent from this discourse, however, is recognition of permanency as a remedy to the exploitation normalized by the Temporary Foreign Worker Program and of the ways in which the TFWP’s exploitative potential comes to be realized through the practices of employers.

## Data Availability

The datasets presented in this article are not available because they are restricted to use by the author through the Research Ethics Approval process.

## Footnotes

1 During the period corresponding to fieldwork, IRCC was called Citizenship and Immigration Canada (CIC).

2 “Four-in four-out” refers to a cumulative-duration rule under the Temporary Foreign Worker Program. The rule specified that temporary foreign workers deemed low-skilled could remain in Canada for a maximum of 4 years, after which they had to remain outside of Canada for four-years prior to re-applying for employment in the country. The rule was taken off the books in 2016.
